# Advances in Mental Time Travel Research in Adolescent Depression: A Narrative Review

**DOI:** 10.31083/AP45509

**Published:** 2025-12-23

**Authors:** Yuan Yuan, Jia-li Liu, Wan-ting Ran, Ya Wang, Gui-fang Chen

**Affiliations:** ^1^Department of Neurology, The Affiliated Hospital of Zunyi Medical University, 563000 Zunyi, Guizhou, China; ^2^Department of Psychiatry, The Affiliated Hospital of Zunyi Medical University, 563000 Zunyi, Guizhou, China; ^3^Key Laboratory of Brain Function and Brain Disease Prevention and Treatment of Guizhou Province, 563000 Zunyi, Guizhou, China; ^4^Neuropsychology and Applied Cognitive Neuroscience Laboratory, Chinese Academy of Sciences Key Laboratory of Mental Health, Institute of Psychology, 100101 Beijing, China; ^5^Department of Psychology, University of Chinese Academy of Sciences, 100101 Beijing, China

**Keywords:** adolescent, autobiographical memory, depression, magnetic resonance imaging, mental time travel

## Abstract

Adolescent depression is a serious public health issue affecting the mental health and quality of life of adolescents worldwide. Mental time travel (MTT), an individual’s capacity to recall the past or look to the future, plays an important role in emotion regulation and mental health. Following the Preferred Reporting Items for Systematic Reviews and Meta-Analyses (PRISMA) guidelines, a systematic literature search was conducted. Due to considerable heterogeneity among the included studies, a narrative synthesis approach was adopted. A total of 22 articles retrieved from PubMed, Web of Science, PsycINFO, and EBSCO (up to October 31, 2024) were included to elucidate the mechanisms underlying MTT impairments and related interventions in depressed adolescents. The main findings indicated that depressed adolescents exhibit overgeneralization of autobiographical memories, impoverished future simulations, and negative bias in MTT constructs. Neuroimaging studies have revealed aberrant activation within the autobiographical memory network, hyperengagement of the self-referential network during MTT tasks, and alterations in emotion regulation circuits. Furthermore, the efficacy of cognitive therapy and memory/imagery-specific training in ameliorating temporal cognitive biases and fostering positive future expectations was demonstrated. These findings underscore the importance of examining adolescent depression through the lens of MTT, offering a promising framework for understanding its cognitive and neural mechanisms and the development of novel intervention strategies.

## Main Points

1. Depressed adolescents with deficits in mental time-travel show overgeneralization of autobiographical memory, a lack of future imagery and a negative memory bias, all of which exacerbate depressive symptoms.

2. Functional Magnetic Resonance Imaging (fMRI) revealed abnormalities in the autobiographical memory network and compensatory activation of self-referential networks. This provides a basis for cognitive training and neuroplasticity interventions.

3. Interventions combining memory training and positive imagery can improve symptoms, but must be tailored to the individual to avoid risks.

## 1. Introduction

Adolescent depression is a psychological disorder that manifests during adolescence. The condition is characterized by symptoms such as low mood, suicidal ideation, anhedonia, irritability, cognitive impairments, sleep and eating disorders, and negative self-evaluation [1]. Its pathogenesis is closely associated with the unique neurodevelopmental characteristics of adolescence. During this period, the brain’s emotional regulation system is underdeveloped, increasing the risk of emotional dysregulation. When considered in conjunction with abnormalities in self-regulation circuits, these factors contribute to the development of depression [2]. Depression has been shown to lead to academic and occupational difficulties, impaired relationships with family and peers, and significant distress and negative thinking. This, in turn, significantly increases the risk of suicide [3]. A review of epidemiological data reveals that the global prevalence of mental disorders among individuals aged 5–24 years is 11.63%. Depression and anxiety disorders are among the top ten causes of disease burden. It is important to note that the prevalence of depression increases with age and has become the primary cause of non-fatal disability in this age group [4, 5]. Adolescence, a critical stage of psychological development, is characterized by the formation of self-identity and the exploration of social roles. This period is also a sensitive window during which psychological issues are more likely to arise [6]. This developmental characteristic renders adolescents more vulnerable to mental health challenges when confronted with factors such as physiological changes, social transitions, and shifts in communication styles. The implications of this vulnerability for their mental health in adulthood are significant [7, 8].

In the process of adolescent psychological adaptation, mental time travel (MTT) is the capacity to revisit past events and to imagine or anticipate future occurrences [9], serving as a crucial mechanism for self-regulation. MTT, proposed by Tulving [10] as a uniquely human cognitive function, comprises the retrieval of autobiographical memory (AM) and the prospective simulation of episodic future thinking (EFT) [11], forming a dynamic temporal coordinate. The former assists individuals in reflecting on and understanding prior experiences, in order to construct self-identity [12]. Additionally, it functions as a regulatory mechanism for emotions [13]. The latter enables individuals to simulate and rehearse future events, thereby contributing to future planning and preparation [14]. Furthermore, it enhances goal motivation and alleviates uncertainty anxiety through positive anticipatory experiences [15, 16, 17]. The central role of MTT in maintaining self-continuity [18], guiding decision-making [19], emotional regulation [18], and social adaptation [20, 21] offers a novel perspective for understanding the cognitive mechanisms underlying adolescent depression.

Although research into the association between MTT and adolescent depression remains in its infancy, evidence has indicated that depressed adolescents exhibit deficits in MTT: difficulties in retrieving episodic memories [22], overgeneralization of autobiographical memory [23], and a lack of positive future thinking [10]. These deficits have the potential to contribute to the development of executive function and emotional-regulation disorders in adolescents [24]. Furthermore, research has demonstrated that MTT can assist adolescents in the development of a coherent self-awareness framework, enhance their sense of meaning and happiness in life, and reduce depressive symptoms [25]. However, the precise mechanism of the relationship between these factors remains to be elucidated. In the investigation of this mechanism, emerging intervention strategies, such as future-specific training and autobiographical memory-retrieval intervention, have been shown to effectively enhance emotional regulation abilities by increasing the specificity and positivity of MTT. This, in turn, disrupts negative cognitive cycles and alleviates depressive symptoms [17, 26]. However, studies have predominantly focused on adult populations. Given the high plasticity of adolescent brains and the rapid development of social cognition, further optimization of MTT intervention training in conjunction with developmental neuroscience may be necessary.

Current empirical studies and meta-analyses have documented impairments in MTT across various clinical populations. For instance, patients with Alzheimer’s disease primarily exhibit difficulties in conceptualizing time [27]; individuals with autism spectrum disorder show reduced detail in AM and EFT [28]; and those with schizophrenia demonstrate deficits in generating specific events and providing detailed descriptions [29]. However, research targeting adolescents with depression remains insufficient. Previous studies have seldom systematically investigated the underlying neural mechanisms or targeted interventions for MTT deficits in this group. This review addresses this gap by systematically synthesizing and analyzing existing literature, with a focus on two key aspects: first, the deficits exhibited by depressed adolescents in MTT, including behavioral manifestations and neural mechanisms of MTT deficits; second, existing intervention measures for depression. In light of these findings, the subsequent discussion focused on potential future research directions.

## 2. Methods

### 2.1 Literature Search and Study Selection

The literature that was searched and retrieved was published prior to October 31, 2024, through PubMed (https://pubmed.ncbi.nlm.nih.gov/), Web of Science (https://www.webofscience.com), PsycINFO (https://www.apa.org/pubs/databases/psycinfo), and EBSCO databases (https://www.ebsco.com/). Search terms were derived from the National Library of Medicine (Medical Subject Headings, MeSH) and its related synonyms. However, since the core concept “mental time travel” is not a MeSH term, synonyms were incorporated into the search strategy to ensure comprehensive literature coverage. The final search strategy utilized Boolean operators (“AND” & “OR”) as follows: (“depression” OR “depressive disorder”) AND (“mental time travel” OR “autobiographical memory” OR “retrospect” OR “autobiographical” OR “remembering past” OR “episodic memory” OR “future thinking” OR “episodic future thinking”) AND (“adolescent” OR “juvenile” OR “teenager”) (Table 1). Due to variations in search strategies and retrievable terms across databases, customized search formulas were developed for each database to enhance precision and effectiveness. Detailed search strategies for each database are provided in **Supplementary Table 1**. Additionally, a manual search of Google Scholar was performed to identify literature not retrieved through the database searches.

**Table 1.** **Search templates, concepts, and terminology**.

Concept 1:	AND	Concept 2:	AND	Concept 3:
Depression		Mental time travel		Age
Depression, depressive disorder		Mental time travel, AM, retrospect, autobiographical, remembering past, episodic memory, future thinking, episodic future thinking		Adolescent, juvenile, teenager

AM, autobiographical memory.

Inclusion criteria for articles were as follows: (a) included at least one empirical study in an area related to MTT (situational memory, episodic future thinking, past, future, autobiographical memory); (b) included individuals diagnosed with depressive disorders using recognized diagnostic criteria, such as the International Statistical Classification of Diseases and Related Health Problems, 10th Revision (ICD-10) criteria or the Diagnostic and Statistical Manual of Mental Disorders, Fifth Edition (DSM-5) criteria for assessing depressive states; (c) included individuals in the age range of early to late adolescence (10–19 years); and (d) published in English.

Exclusion criteria were as follows: (a) no explicit diagnosis of depressive disorders and no explicit reference to MTT-related content; (b) age out of range; and (c) non-full text, as well as studies in reviews, meta-analyses, research letters, and commentaries. Among the articles included in the analysis, articles using magnetic resonance/functional neuroimaging were further analyzed. The research screening process was conducted independently by two authors (YY and WTR) according to the inclusion and exclusion criteria. Any disagreement between the authors during assessment was resolved through structured discussion. Where consensus could not be reached, a third author (GFC) was consulted to make the final decision.

### 2.2 Data Extraction and Quality Assessment

The following data were extracted from the included studies: basic information (first author, year of publication, study type); participant characteristics (age, diagnosis, sample size, percentage of women, etc*.*); MTT tasks; questionnaires/scales; effect sizes; and main findings. To assess the potential impact of study quality on the conclusions of the review, the quality of the included studies was assessed using the Mixed Methods Appraisal Tool (MMAT, version 2018, http://mixedmethodsappraisaltoolpublic.pbworks.com) [30]. Each research paper was initially screened to determine whether it presented a clear research question and whether the collected data adequately addressed that question. Subsequently, appropriate study categories were selected to evaluate five corresponding design-specific criteria. Each criterion was rated as “Yes”, “No”, or “Can’t tell”. Items rated “Yes” were assigned a score of 1, and those rated “No” or “Can’t tell” (indicating insufficient reporting in the text) received a score of 0. Total scores ranged from 0 to 5, with 5 representing the highest quality. Two authors independently completed the scoring, resolving disagreements through discussion until consensus was reached.

## 3. Results

### 3.1 Literature Screening Process and Characteristics of Included Studies

**Fig. 1.**
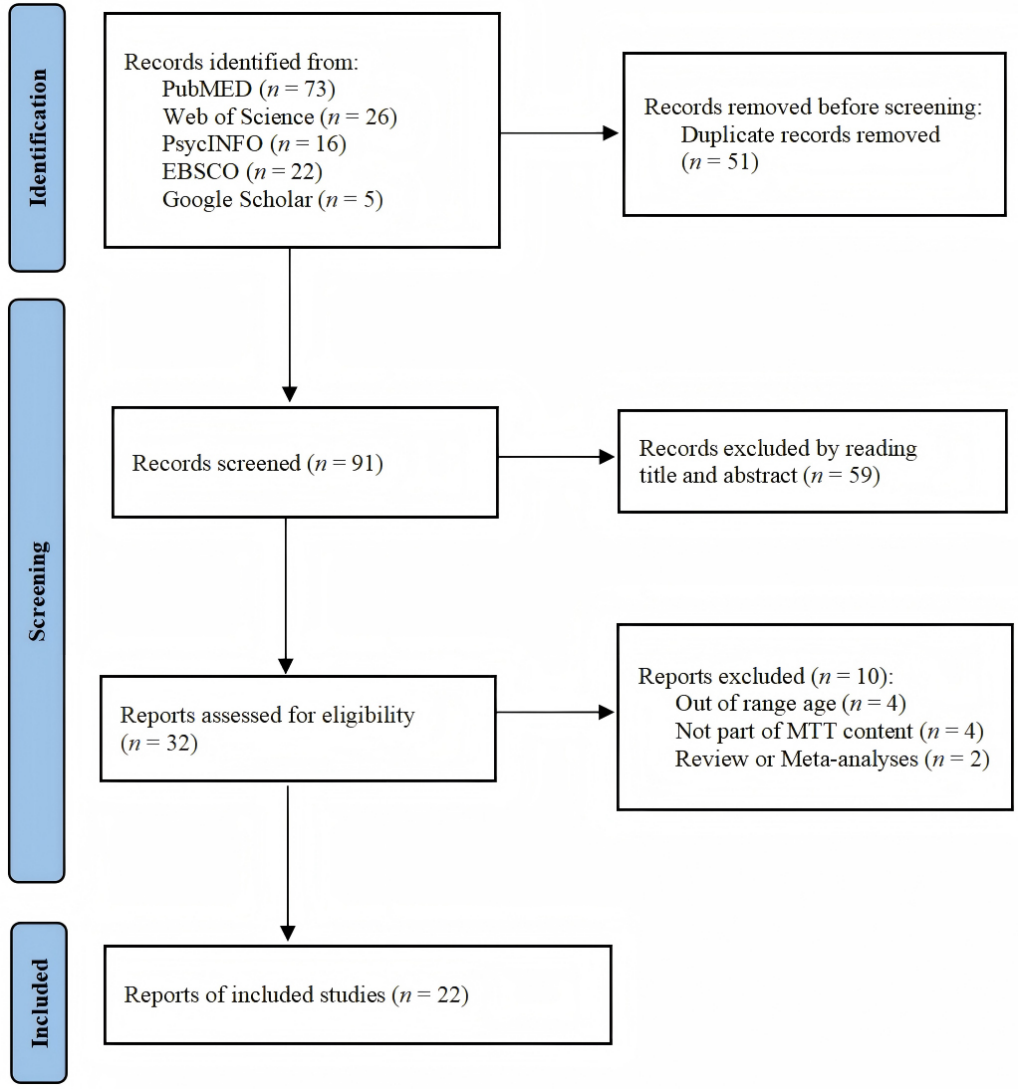
**Flow diagram of the article selection process**.

We initially searched 142 articles, of which 73 were retrieved from PubMED, 26 from Web of Science, 16 from PsycINFO, 22 from EBSCO, and 5 from Google Scholar. Duplicate checking for de-duplication through EndNote left 91 articles, and 59 were excluded by reading the title and abstract. Full-text screening of the remaining 32 articles led to the exclusion of 10 additional studies, resulting in 22 articles ultimately included in this review’s comprehensive analysis. The Preferred Reporting Items for Systematic Reviews and Meta-Analyses (PRISMA) flowchart (see Fig. 1) was adopted to standardize the literature screening process.

Basic data for all included studies are detailed in Table 2 (Ref. [1, 2, 23, 24, 31, 32, 33, 34, 35, 36, 37, 38, 39, 40, 41, 42, 43, 44, 45, 46, 47, 48]). Among the 22 final included articles (comprising 23 independent studies), study types included 1 qualitative study, 2 quantitative randomized controlled trials, and 19 non-randomized quantitative studies. Systematic quality assessment revealed that 7 studies scored 5 points, 14 scored 4 points, and 2 scored 3 points (see **Supplementary Tables 2–4** for details). This indicates that the overall study quality was acceptable.

**Table 2.** **Selected articles for MTT for adolescent depression (*n* = 22)**.

Author (publication year)	Study type	Sample size	Age	Sex ratio (% of women)	Diagnosis	MTT tasks	Time direction	Questionnaire/Scale	Effect size	Main findings	MMAT score
Rawal and Rice (2012) [23]	⑤	55	10–18	59.60%	Depression	AMT	Remembering the past	WISC;	*OR* = 1.65	Overgeneralized negative memories predict new-onset depressive symptoms	3
								CRSS;			
								LEC			
Hamlat *et al*. (2015) [39]	⑤	160	12–13	43.80%	Depression	modified AMT	Remembering the past	CDI;	*r* = 0.48	Depressive symptoms were positively associated with rumination (*p* < 0.001)	4
								CRSQ;			
								ALEQ;	*r* = 0.27	Depressive symptoms were positively associated with negative life events (*p* < 0.001)	
								CLES			
Hards *et al*. (2024) [1]	⑥	584	13–18	52.40%	Depression	‘I Will Be’ task	Imagine the future	MFQ	*r* = –0.16	Depression severity negatively correlated with potential self-potency (*p* < 0.001)	5
Hawkins-Elder and Salmon (2020) [35]	⑤	554	10–14	59.20%	Depression	Mi - AMT	Remembering the past	ARS;	*r* = 0.13	Observer perspective positively associated with cross-sectional depression (*p* < 0.001)	5
								CDI-2 SR			
Sumner *et al*. (2011) [41]	⑤	55	16–18	75%	MDD	AMT	Remembering the past	IDD;	*OR* = 0.08	Proportion of specific memories predicts onset of depression with high chronic interpersonal stress	5
								LSI	*OR* = 0.22	Specific memory ratios interact with chronic interpersonal stress	
Salmon *et al*. (2021) [37]	⑤	132	14–18	65%	Depression	VFT;	Remembering the past	CDI-2	β = 0.20	Plot details were significantly and positively associated with depressive symptoms (*p* < 0.01)	5
						Mi - AMT					
Quevedo *et al*. (2020)^a^ [2]	④	53	mean: 16.11	67.90%	Depression	Recalling positive AM for a neurofeedback task	Remembering the past	K-SADS-PL;	*r* = 0.307	Right prefrontal-amygdala correlated positively with changes in depression (*p* < 0.05)	4
								CDRS			
Champagne *et al*. (2016) [31]	⑥	65	11–18	60%	MDD	AMT	Remembering the past	K-SADS-PL;	*η²* = 0.22	Never depressed adolescents had significantly more specific memory recall	4
								CDI			
van Houtum *et al*. (2023)^a^ [45]	④	69	11–17	66.70%	MDD	RAM-task	Remembering the past	K-SADS-PL;	*d* = –0.62	Low pleasure and vividness recall are associated with increased network activation	5
								PHQ-9			
Ahrweiler *et al*. (2022)^a^ [46]	③	53	mean: 16.08–16.26	67.90%	Depression	Recalling positive AM for a neurofeedback task	Remembering the past	K-SADS-PL;	*d* = –0.61	Significant reduction in depressive symptoms before and after neurofeedback scans	4
								CDRS;			
								WASI			
Tang *et al*. (2023) [36]	①	19	16–19	84.20%	Depression	Semi-structured interviews	Imagine the future	K-SADS-PL;	/	Depression and anxiety significantly reduce future thinking and increase negative expectations	5
								CDRS			
Lakshmi *et al*. (2024) [43]	⑥	57	13–17	73.70%	MDE	EFT-T;	Imagine the future	RCADS;	*r* = –0.52	Expected happiness is negatively associated with depressive symptoms	4
						AMT		TEPS;			
								WISC-IV	*r* = –0.43	Consumptive pleasure is negatively associated with depressive symptoms	
Park *et al*. (2002) [33]	④	155	12–17	65.80%	MDD	AMT	Remembering the past	K-SADS-PL;	*d* = –0.78	Categorical memory for negative cues outnumbered positive cues in full-depression	4
								HAMD;			
								WISC-II;			
								MFQ			
Park *et al*. (2004) [40]	②	134	12–17	67.90%	MDD	AMT	Remembering the past	K-SADS-PL;	*d* = –0.51	Induced rumination increases depression	5
								HAMD;			
								WISC-II;			
								MFQ;	*d* = –0.983	Induced rumination increases negative AM	
								VAS			
Park *et al*. (2005) [42]	⑤	94	12–17	70.00%	MDD	AMT	Remembering the past	K-SADS-PL;	*OR* = 1.09	Self-depreciating sexual experiences predict persistent depression in adolescents with first-episode MDD	4
								HAMD;			
								WISC-III;			
								MFQ;	*r* = 0.27	Self-destructive sexual experiences are significantly associated with HAMD (*p* < 0.01)	
								RSQ;			
de Jong-Meyer *et al*. (2007) [47]	②	48	16–18	72.90%	Depression	VFT;	Imagine the future	BDI;	*η²* = 0.36	Significant interactions between mood potency and mood induction	4
						FTT		BAI			
Holt *et al*. (2016)^a^ [44]	⑥	86	11–17	80.20%	MDD	Retrieval of encoded emotional memory	Remembering the past	EHI;	*d* = 0.66	Abnormal medial temporal and prefrontal lobe activation during emotional memory encoding in depressed adolescents	4
								K-SADS-PL;			
								state/trait anxiety inventory			
Pile and Lau (2018) [48]	⑥	369	11–16	54.10%	Depression	PIT	Imagine the future	RIES-C;	*η²* = 0.063	More severe events are associated with more depressive symptoms	4
								SCARED;			
								CDI			
Warne *et al*. (2020) [38]	⑤	4111	12.5–16.5	/	Depression	AMT	Remembering the past	sMFQ	*r* = 0.112	Overgeneralized negative AM was associated with baseline depressive symptoms (*p* < 0.001)	4
									*r* = 0.116	Overgeneralized negative AM was associated with follow-up depressive symptoms (*p* < 0.001)	
Kuyken and Howell (2006) [34]	⑥	65	12–18	78.50%	MDD	AMT;	Remembering the past	BDI‐II;	*d* = 0.54	The currently depressed group retrieved more recent memories than the once depressed group	3
						VFT		THQ;			
								CIES	*d* = 0.72	The depressed group recalled negative memories more frequently	
Kuyken *et al*. (2006) [24]	⑥	62	12–18	80.60%	MDD	AMT;	Remembering the past	BDI - II;	*d* = 1.26	Depressed non-trauma group overgeneralized significantly more than never depressed group	4
						VFT		THQ;			
								CIES - 8			
Kuyken and Dalgleish (2011) (Study 1) [32]	⑥	179	14–18	62%	MDD	AMT;	Remembering the past	PHQ-A;	*r* = 0.16	Neuroticism was significantly associated with negative cued word category memory (*p* < 0.03)	4
						VFT		EPQ-N;			
								BDI-II	*r* = 0.70	Neuroticism was significantly associated with BDI scores (*p* < 0.001)	
Kuyken and Dalgleish (2011) (Study 2) [32]	④	30	14–18	77%	MDD	AMT;	Remembering the past	PHQ-A;	*OR* = 12.25	Risk of depression is associated with categorical memory that tends to retrieve negative cue words	4
						VFT		EPQ-N;			
								BDI-II			

*Note*: ^a^ refers to the use of functional magnetic resonance imaging in the article. Study type: ①Qualitative study; ②Randomized controlled clinical trial; ③Non-randomized controlled trials; ④Case-control study; ⑤Longitudinal study; ⑥Cross-sectional analytic study. AMT, Autobiographical Memory Test; EFT-T, Episodic future thinking task; FTT, Future Thinking Task; Mi-AMT, Minimal Instructions Autobiographical Memory Test; VFT, Visual analogue scales. ALEQ, Adolescent Life Events Questionnaire; ARS, Affect Regulation Scale; BAI, Beck Anxiety Inventory; BDI, Beck Depression Inventory; CDI, Children’s Depression Inventory; CDRS, Continuous Children’s Depression Rating Scale; CIES, Children’s Impact of Event Scale; CLES, Children’s Life Events Scale; CRSQ, Children’s Response Styles Questionnaire; CRSS, Children’s Response Styles Scale; EHI, Edinburgh Handedness Inventory; EPQ-N, Eysenck Personality Questionnaire-Revised Neuroticism sub-scale; HAMD, Hamilton Depression Rating Scale; IDD, Inventory to Diagnose Depression; K-SADS-PL, Kiddie Schedule for Affective Disorders and Schizophrenia Present and Lifetime Version; LEC, Life Events Checklist; MFQ, Mood and Feelings Questionnaire; PHQ-9/A, Patient Health Questionnaire-9/A; PIT, Prospective Imagery Task; QIDS, Quick Inventory of Depressive Symptomatology; RAM-task, ‘reliving autobiographical memories’ -task; RCADS, Revised Child Anxiety and Depression scale; RIES-C, Revised Impact of Event Scale, child version; RSQ, Ruminative Response style Questionnaire; SIQ, Suicidal Ideation Questionnaire; sMFQ, short Mood and Feelings Questionnaire; TEPS, Temporal Experience of Pleasure Scale; THQ, Trauma History Questionnaire; VAS, Visual analogue scales; WASI, Wechsler Abbreviated Scale Intelligence; WISC-IV, Wechsler’s Intelligence Scale for Children, 4th Ed; MMD, Major Depressive Disorder; MDE, Major Depressive Episode; MMAT, Mixed Methods Appraisal Tool.

### 3.2 MTT’s Behavioral Studies

Current research on depressive individuals’ recollection of past events predominantly uses autobiographical-memory-task paradigms. That paradigm requires participants to retrieve autobiographical memories based on cue words, subsequently encoding the recalled content into one of five categories: concrete, expanded, categorical, semantically related, or absent [49]. Studies revealed that depressed adolescents exhibit the following memory characteristics in autobiographical memory tasks: (a) Information retrieval and recall: This is characterized by overgeneralization of autobiographical memories [23, 24] and reduced generation of specific memories [31]. For instance, participants were only able to produce general statements, such as “I went out to play with my friends”, and experienced difficulty retrieving memory content that contained specific temporal details and situational elements, such as “Last month, on a weekend afternoon, I went to an amusement park with my friends and rode the roller coaster, bumper cars, and other attractions; we all had a great time”. (b) Emotional bias: Negative memory and emotional bias are present [32], manifested as more categorized memories of negative cues than positive cues (*z* = –2.5, *p* < 0.05). Negative memory bias is positively correlated with Hamilton Depression Rating Scale scores (*r* = 0.270, *p* < 0.05) [33], suggesting that emotional memory bias may serve as a potential cognitive marker for the severity of depression. (c) Coherence: The integrity of autobiographical memory coherence is diminished, as evidenced by the participants’ difficulty in constructing a comprehensive timeline of events [50]. (d) Perspective: The proportion of observer-perspective memory increases, meaning that individuals tend to reconstruct events from a third-person perspective during recall, and observer perspective is significantly correlated with depressive symptoms (*r* = 0.13, *p* < 0.001) [34, 35]. Those findings suggested that deficits in MTT may impair the ability of depressed adolescents to retrieve and process memory cues, diminish their capacity for emotion regulation, and reduce their self-awareness and sense of identity. Previous research has demonstrated that adolescent depression has the potential to impair cognitive function and result in specific episodic-memory impairments [22]. That suggests that patients may be more prone to retrieving negative or unpleasant memory content. The phenomenon of memory-retrieval bias has been linked to tendencies of excessive self-reflection and self-blame [36]. When depressed adolescents are prompted to recall past events, they may re-experience the emotional states associated with negative memories. That has been shown to exacerbate and prolong depressive symptoms [37]. Furthermore, their capacity to re-experience positive emotions may also be diminished [38, 50]. That persistent negative memory retrieval pattern may form a vicious cycle, further exacerbating depressive symptoms [51].

Additionally, the overgeneralization of autobiographical memory in depressed adolescents interacts with the following factors: (a) Life stress events and rumination: Depressed adolescents with high rumination tendencies exhibit increased levels of overgeneralization of autobiographical memory under the influence of life stress events, with female adolescents being more susceptible than males to autobiographical memory overgeneralization due to rumination [39]. Research has also found that inducing rumination increases negative emotions and excessive generalization of negative memory cues in depressed adolescents [40]. (b) Chronic interpersonal relationships: As an important environmental risk factor, elevated levels of chronic interpersonal stress can increase the risk of depressive symptoms through excessive generalization of autobiographical memory [41]. (c) Self-perception biases: Overgeneralization of positive autobiographical memories in depressed individuals was found to be associated with self-deprecating experiences (*r* = 0.47, *p* < 0.001) [42], manifested as avoiding in-depth recall of specific events through generalized positive memories while maintaining negative self-perceptions. These interactive mechanisms play a predictive and promotional role in the onset, persistence, and exacerbation of depressive symptoms.

Depression has also been shown to diminish adolescents’ capacity and motivation to imagine the future, thereby influencing the configuration, vividness, and valence of imagined future events [36]. For instance, depressed adolescents exhibit a negative bias toward future thinking, with their imagined content filled with despair and pessimism [52]. Some individuals avoid thinking about the future or are unable to imagine it at all [36], leading to a reduction in the vividness of positive future imagery among adolescents. Anticipated pleasure was found to be negatively correlated with depressive symptoms (*r* = –0.52, *p* < 0.001), meaning that reduced pleasure may exacerbate depressive symptoms. That negative future thinking interacts with depression, serving as mutual predictive factors [1]. EFT impairments further affect adolescents’ self-processing (damaging self-continuity), executive function (weakening the ability to set and plan for future goals), and reward processing (reducing sensitivity to anticipated pleasure) [43], thereby influencing their decision-making regarding future events and problem-solving approaches. Reduced positive future thinking plays a pivotal role in the perpetuation of adolescent depression and has been shown to exacerbate the development of suicidal ideation [53, 54]. In the Hards *et al*. study [1], adolescents were tasked with envisioning their future selves, thereby engaging in a form of future-oriented thinking. The results indicated that adolescents with more severe depression were more likely to generate a greater number of negative “possible selves”. However, it is noteworthy that adolescents with varying degrees of depression all generated positive possible selves. That finding suggested that adolescents with depression continue to harbor positive expectations regarding their future.

### 3.3 MTT’s Neural Mechanisms Study

According to the preceding examination of the behavioral characteristics of AM and EFT in depressed adolescents, functional magnetic resonance imaging (fMRI) studies have provided evidence elucidating the neural mechanisms underlying MTT dysfunction within that sample. Findings indicated that MTT dysfunction in depressed adolescents correlated with abnormal activation patterns within the autobiographical memory network (AMN). That network includes key regions such as the medial prefrontal cortex (mPFC), posterior cingulate cortex (PCC), and temporoparietal junction (TPJ), with core functions in self-referential processing and the retrieval and simulation of episodic memories [44, 55]. Although the AMN and DMN exhibit significant overlap, they are not identical. Abnormal DMN activity may not only reflect impairments in MTT but has also been implicated in other psychological processes such as rumination and social cognition [56]. Therefore, interpretation of DMN dysfunction must consider the contributions of multiple cognitive and affective processes.

A study using event-related independent component analysis (eICA) showed that adolescents with depression exhibited activation in the AMN when recalling positive AM. When recalling memories with low pleasantness and low vividness, brain regions associated with self-referential processing (e.g., mPFC and PCC) showed enhanced activation. That may correlate with the diminished sense of self-worth commonly observed in adolescent depression. Notably, no significant differences in neural activity were observed between depressed adolescents and healthy controls during retrieval of positive memories, suggesting that depressed adolescents may still retain responsiveness to positive emotional stimuli [45].

A real-time fMRI neurofeedback study demonstrated that adolescents with depression reported reduced depressive and ruminative symptoms after a task modulating amygdala-hippocampal complex (AMYHIPP) activity by recalling positive autobiographical memories. Generalized linear model analysis indicated that symptom improvement correlated with increased activation in self-referential networks [46]. Another neurofeedback study, based on seed-point functional connectivity analysis, found that enhanced connectivity between the right amygdala and prefrontal cortex during positive AM recall was positively correlated with symptom improvement (r = 0.307, *p* < 0.05), suggesting that this pathway may play a role in the neurobiological mechanisms of symptom remission [2].

In summary, functional impairments in depressed adolescents during the MTT may involve abnormal activation patterns in the AMN, excessive activation of the self-referential network, and alterations in emotional regulation circuits. Future research should further elucidate the mechanisms underlying different brain networks during the MTT by distinguishing task-related from resting-state functional characteristics and integrating multiple analytical approaches.

### 3.4 MTT — Related Intervention Mechanisms

Deficits in past and future thinking in adolescent depression are considered an important characteristic and should be targeted for intervention and improvement [43]. With respect to the recollection of the past, cognitive-recollection therapy can guide individuals to reconstruct their perceptions of past events through integrative recollection and to redefine past experiences through instrumental recollection [57]. That approach shows promise in enhancing self-efficacy and sense of meaning in life among depressed adolescents, thereby fostering a more positive future orientation [26].

Memory-specificity training uses a behavioral-training paradigm [58] involving repeated practice in retrieving specific memories in response to positive cue words. It may effectively reduce persistent negative memory retrieval and alleviate future sadness [59]. However, that method may be more suitable for those depressed adolescents with high baseline levels of negative memories, and individualized application should be considered.

In terms of imagining the future, intervening with depressed adolescents to generate positive EFT is also an important intervention measure [60]. Future-specificity training, adapted from memory-specificity training, involves using cue words to imagine neutral or positive future events. That technique seems to enhance the detail and emotional vividness of positive EFT and reduce avoidance of positive emotional experiences [61]. Preliminary evidence has indicated that such training may influence the specificity, detail level, and imagery of EFT, thereby enhancing the ability to imagine future events [17] and increasing the evocation of anticipated pleasure, which can effectively alleviate anhedonia and improve depressive symptoms [61].

Another approach is the emotion-induction paradigm, which requires participants to listen to classical music that evokes positive or negative emotions while imagining corresponding events. Research has found that the emotion value of the positive-emotion-induction group increased and their symptoms were alleviated, suggesting that this method may hold potential intervention value [47].

The connection between past and future thinking is inseparable. Therefore, a comprehensive treatment approach is needed to achieve effective therapeutic outcomes for both. For instance, in the Bogaert *et al*. study [51], they used a combination of positive-event training and past and future autobiographical thinking, which led to substantial improvements in AM and EFT at the conclusion of the training program and during a two-month follow-up period. Additionally, there was a notable enhancement in anhedonia. Those studies underscore the pivotal role of positive events [48].

Although the above findings suggest promising prospects for adolescent-depression interventions, several considerations warrant attention. First, many studies featured small sample sizes and often lacked active control groups, thereby limiting the reliability of conclusions. Second, most intervention protocols were initially developed and validated in adult samples; their efficacy and safety in adolescents require further rigorous assessment to ensure precise adaptation. Most critically, overly optimistic future thinking may increase feelings of hopelessness and suicide risk among adolescents with major depressive disorder [54], which suggests that such techniques should be applied with caution. Therefore, future intervention strategies should account for individual differences among depressed adolescents. To enhance treatment outcomes and effectively address the diverse needs of adolescent patients, it is recommended that tailored approaches are developed.

## 4. Discussion 

This review summarized deficits in MTT exhibited by depressed adolescents, including overgeneralized autobiographical memory, reduced memory coherence, preference for negative emotional content, and impaired future-simulation ability. Neuroimaging evidence further revealed the underlying abnormalities in the neural mechanisms behind these deficits, such as dysregulation of autobiographical-memory-network activation, involvement of self-referential processing regions, and alterations in emotional regulation circuits. Research has also indicated that memory and future-specific training can effectively alleviate depressive symptoms, suggesting that MTT-based interventions hold potential for clinical translation.

### 4.1 Heterogeneity Among Included Studies and Its Implications 

Although this study employed a systematic review methodology for literature retrieval and screening, the selected papers exhibited high degree of heterogeneity, including differences in study design, participant samples, experimental paradigms, and outcome measures. Conducting a meta-analysis (quantitative synthesis) under these circumstances could yield misleading results. Therefore, we ultimately adopted a narrative synthesis approach to integrate and interpret the research findings. This method allowed for a comprehensive examination of the evidence, strengths, and limitations presented in the included studies while avoiding potentially misleading pooled-effect sizes.

The studies included in this review exhibited significant heterogeneity, necessitating careful consideration when interpreting results: (a) participants exhibited varying degrees of depressive symptoms (e.g., major depressive disorder, major depressive episode, or depressive state). Differences in depression severity may correlate with the degree of MTT dysfunction, although this association was not formally tested in this review. Future research should compare MTT performance across different levels of depression severity. (b) All included participants were adolescents aged 12–19 years, a period of critical brain development. Differences in cognitive function between early and late adolescence may have influenced MTT behavioral and neural mechanisms. Future studies should stratify participants into early, middle, and late adolescence to explore developmental trajectories of MTT-related cognitive patterns. (c) Adolescent females exhibited higher rates of depressive symptoms and diagnoses [62]. Thus, sex may modulate MTT performance. Future studies should strive for sex balance and use more refined MTT assessments. (d) Comorbid conditions (e.g., anxiety) and treatment status represent significant confounding variables. For instance, anxiety may have exacerbated deficits in simulating future events [48], thereby intensifying MTT dysfunction. Similarly, differences in pharmacological or psychotherapeutic interventions may have influenced MTT-related behavioral and neuroimaging outcomes among depressed adolescents.

The behavioral and neuroimaging findings in this review should be understood as the result of multiple interacting factors rather than attributed to any single cause. Although this significant heterogeneity posed challenges for analyzing and interpreting current results, it also underscored the need for future studies to use more rigorous designs (incorporating these variables as covariates) to elucidate the underlying mechanisms of MTT.

### 4.2 Limitations and Future Directions 

Despite significant progress, the recent literature exhibited several limitations: (a) Methodological constraints: Sex imbalance (predominantly female participants), small sample sizes (some studies with *n* < 30), and reliance on self-report measures may have limited the statistical power and introduced bias. The scarcity of longitudinal studies and brief follow-up periods further restricted understanding of MTT developmental trajectories. (b) Incomplete explanation of neural mechanisms: The interactive mechanisms across multiple brain regions remain unclear. Additionally, insufficient motor control in fMRI data may have compromised the accuracy of functional connectivity analyses. (c) Study design: The evidence partly stemmed from cross-sectional studies, making it difficult to draw definitive conclusions about the directional relationship between MTT deficits and adolescent depression. Depression and MTT deficits may have influenced each other reciprocally, or other potential factors could have simultaneously contributed to both depressive episodes and MTT dysfunction. Therefore, future studies should use longitudinal or intervention designs to clarify causal relationships. (d) This review exclusively included English-language literature, potentially introducing language bias by excluding studies in other languages. Future research should broaden language inclusion to address this limitation.

Future research should prioritize the following directions: (a) Optimizing study design: Conduct longitudinal cohort studies using larger, well-characterized samples with balanced sex and age distribution. Supplement self-report data with structured interviews and objective cognitive assessment tools. (b) Technological breakthroughs: Apply multimodal neuroimaging techniques (e.g., resting-state fMRI combined with diffusion tensor imaging) to elucidate structure-function relationships within key brain networks. Optimize motor control and data preprocessing workflows. (c) Clinical translation: Develop personalized intervention protocols centered on MTT, such as VR-based simulated scenario training or AI-guided immersive training. Integrate neurofeedback technologies to assess and promote neuroplasticity changes.

Advances in this field may aid in the early identification and intervention of adolescent depression: indicators such as the precision of future scenario simulations and neurobiological markers (e.g., prefrontal-amygdala connectivity) can serve as objective assessments of depression risk. Modulating time-perception biases through MTT training can compensate for limitations in pharmacological treatments. Integrating MTT training into school mental health programs not only enhances temporal cognition but also provides support for early intervention.

## 5. Conclusions

This review summarized previous studies related to MTT in depressed adolescents and identified associations between core cognitive deficits, such as overgeneralization of autobiographical memories and decreased ability to simulate future scenarios, and abnormalities in the functioning of specific brain networks. These findings reveal the potential of MTT as a mechanism-oriented therapeutic intervention. Although memory-specific training and positive future imagination techniques demonstrate therapeutic efficacy, their application must be carefully personalised, with particular attention paid to the safety and efficacy of interventions for adolescents at risk of suicide. Future research should use large-sample longitudinal designs with covariate-controlled assessments to enhance the validity of inferences. Multimodal brain-imaging techniques may be used to elucidate the structural and functional mechanisms of brain networks. Finally, personalized intervention programs based on MTT not only provide objective biomarkers for early detection and risk assessment, but also support the implementation of targeted school and clinic strategies to modify cognitive biases.

In summary, investigating adolescent depression through the lens of mental time-travel offers a promising framework for advancing clinical insight and intervention, ultimately contributing to the improvement of mental health outcomes in this population.

## Supplementary Material

Supplementary material associated with this article can be found, in the online version, at https://doi.org/10.31083/AP45509.
