# Supplementary material for: Advances in Mental Time Travel Research in Adolescent Depression: A Narrative Review
Source: Alpha Psychiatry. 2025 Dec 23;26(6):45509. doi: 10.31083/AP45509 (PMC12781217; doi:10.31083/AP45509)
Supplement: Supplementary file 1 [file 2757-8038-26-6-45509-s1.docx]

**Advances in Mental Time Travel Research in Adolescent Depression: A Narrative Review**

**Supplementary Material**

**Supplementary Table 1** Search formulas corresponding to the four databases

| Datebase | Retrieval formula |
| --- | --- |
| PubMED | ((depression[Title/Abstract] OR depressive disorder[Title/Abstract]) AND (Mental time travel[Title/Abstract]OR AM[Title/Abstract] OR retrospect[Title/Abstract] OR autobiographical[Title/Abstract] OR remembering past[Title/Abstract] OR episodic memory[Title/Abstract] OR future thinking[Title/Abstract] OR episodic future thinking[Title/Abstract])) AND (adolescent[Title/Abstract] OR juvenile[Title/Abstract] OR teenager[Title/Abstract]) |
| Web of Science | depression OR depressive disorder (Title) and Mental time travel OR AM OR retrospec OR autobiographical OR remembering past OR episodic memory OR future thinking OR episodic future thinking (Title) and adolescent OR juvenile OR teenager (Title) |
| PsycINFO | TI (depression OR depressive disorder) AND TI (autobiographical OR remembering past OR episodic memory OR future thinking OR episodic future thinking) AND TI (adolescent OR juvenile OR teenager) |
| EBSCO | TI (depression OR depressive disorder) AND TI (Mental time travel OR AM OR retrospect OR autobiographical OR remembering past OR episodic memory OR future thinking OR episodic future thinking) AND TI (adolescent OR juvenile OR teenager) |

**Supplementary Table 2** Non-randomized quantitative study MMAT scores

| author | Year | S1. Are there clear research questions? | S2. Do the collected data allow to address the research | 1.Are the participants representative of the target population? | 2.Are measurements appropriate regarding both the outcome and intervention (or exposure)? | 3.Are there complete outcome data? | 4.Are the confounders accounted for in the design and analysis? | 5.During the study period, is the intervention administered (or exposure occurred) as intended? |
| --- | --- | --- | --- | --- | --- | --- | --- | --- |
| Adhip Rawal et al. | 2012 | Yes | Yes | Yes | Yes | No | Yes | Can't tell |
| Elissa J Hamlat et al. | 2014 | Yes | Yes | Yes | Yes | No | Yes | Yes |
| Emily Hards et al. | 2024 | Yes | Yes | Yes | Yes | Yes | Yes | Yes |
| Hannah Hawkins-Elder et al. | 2020 | Yes | Yes | Yes | Yes | Yes | Yes | Yes |
| Jennifer A. Sumner et al. | 2010 | Yes | Yes | Yes | Yes | Yes | Yes | Yes |
| Karen Salmon et al. | 2020 | Yes | Yes | Yes | Yes | Yes | Yes | Yes |
| Karina Quevedo et al. | 2020 | Yes | Yes | Yes | Yes | Can't tell | Yes | Yes |
| Katelynn Champagne et al. | 2016 | Yes | Yes | Yes | Yes | Can't tell | Yes | Yes |
| Lisanne A.E.M. van Houtum et al. | 2023 | Yes | Yes | Yes | Yes | Yes | Yes | Yes |
| Natasha Ahrweiler et al. | 2022 | Yes | Yes | Can't tell | Yes | Yes | Yes | Yes |
| Pooja M Lakshmi et al. | 2023 | Yes | Yes | Yes | Yes | Can't tell | Yes | Yes |
| R.J. Park et al. | 2002 | Yes | Yes | Yes | Yes | Yes | Yes | Can't tell |
| R.J. Park et al. | 2005 | Yes | Yes | Yes | Yes | Yes | Yes | Can't tell |
| Rosemary J. Holta et al. | 2016 | Yes | Yes | Yes | Yes | Yes | Yes | Can't tell |
| Victoria Pile et al. | 2018 | Yes | Yes | Yes | Yes | No | Yes | Yes |
| Warne N et al. | 2020 | Yes | Yes | Yes | Yes | No | Yes | Yes |

**Table2** (continued)

| author | Year | S1. Are there clear research questions? | S2. Do the collected data allow to address the research | 1.Are the participants representative of the target population? | 2.Are measurements appropriate regarding both the outcome and intervention (or exposure)? | 3.Are there complete outcome data? | 4.Are the confounders accounted for in the design and analysis? | 5.During the study period, is the intervention administered (or exposure occurred) as intended? |
| --- | --- | --- | --- | --- | --- | --- | --- | --- |
| Willem Kuyken et al. | 2006 | Yes | Yes | Yes | Yes | No | Yes | Can't tell |
| Willem Kuyken et al. | 2006 | Yes | Yes | Yes | Yes | Yes | Yes | Can't tell |
| Willem Kuyken et al.（study1） | 2011 | Yes | Yes | Yes | Yes | Yes | Yes | Can't tell |
| Willem Kuyken et al.（study2） | 2011 | Yes | Yes | Yes | Yes | Yes | Yes | Can't tell |

**Supplementary Table 3** Quantitative Randomized Controlled Trial MMAT Score

| author | Year | S1. Are there clear research questions? | S2. Do the collected data allow to address the research | 1.Is randomization appropriately performed? | 2.Are the groups comparable at baseline? | 3.Are there complete outcome data? | 4.Are outcome assessors blinded to the intervention provided? | 5.Did the participants adhere to the assigned intervention? |
| --- | --- | --- | --- | --- | --- | --- | --- | --- |
| R.J. Park et al. | 2004 | Yes | Yes | Yes | Yes | Yes | Yes | Yes |
| Renate de Jong-Meyer et al. | 2007 | Yes | Yes | Yes | Yes | Yes | No | Yes |

**Supplementary Table 4** Qualitative research MMAT scores

| author | Year | S1. Are there clear research questions? | S2. Do the collected data allow to address the research | 1.Is the qualitative approach appropriate to answer the research question? | 2.Are the qualitative data collection methods adequate to address the research question? | 3.Are the findings adequately derived from the data? | 4.Is the interpretation of results sufficiently substantiated by data? | 5.Is there coherence between qualitative data sources, collection, analysis and interpretation? |
| --- | --- | --- | --- | --- | --- | --- | --- | --- |
| Peiyao Tang et al. | 2023 | Yes | Yes | Yes | Yes | Yes | Yes | Yes |
